# Analyzing the Bioactivity of a Novel Bone Cement: An In Vitro Study

**DOI:** 10.7759/cureus.68422

**Published:** 2024-09-01

**Authors:** Janani Balachandran, Kalai Vani, Venkatesh Alagarsamy, Pooraninagalakshmi J, Jembulingam Sabarathinam

**Affiliations:** 1 Department of Conservative Dentistry and Endodontics, Sree Balaji Dental College & Hospital, Chennai, IND

**Keywords:** mineral trioxide aggregate (mta), hydroxyapatite, inductively coupled plasma atomic spectoscopy, bone cement, bioactive

## Abstract

Aim: To analyze the effect of bioactive bone cement (BBC) placed in a phosphate buffer saline solution in comparison to mineral trioxide aggregate (MTA).

Methodology: Ten samples each of BBC (group 1) and MTA (group 2) were prepared and stored in a phosphate buffer saline solution. After three days of storage, white precipitates were formed on the surface of the samples. The solution with precipitates from each sample was analyzed for the presence of calcium and phosphate ions with coupled plasma atomic spectroscopy.

Results: BBC showed a significant amount of calcium and phosphate release after a seven-day storage period in phosphate buffer saline solution. Calcium release was significantly higher in group 1 (MTA) (p < 0.001) compared to that in group 2 (BBC), while group 2 (BBC) (p < 0.001) exhibited greater phosphate release compared to group 1 (MTA).

Conclusion: BBC (group 2) retains its bioactivity when it comes into contact with a stimulated oral environment (STF). This demonstrates that BBC is bioactive in a simulated oral environment. Moreover, it retained good handling properties and could be easily manipulated into a dough form. Clinically, in cases of apical surgery, internal resorption or perforation repair where material placement poses difficulty, BBC will prove to be beneficial.

## Introduction

The dawn of regenerative biomaterials has propelled the field of endodontics into new realms. Regenerative endodontics is defined as “biologically based procedures designed to replace damaged structures, including dentin and root structures, as well as cells of the pulp-dentin complex” [1]. Mineral trioxide aggregate (MTA) is one such material that exhibits remarkable bioactivity, along with other favorable properties, such as biocompatibility [2], antibacterial activity [3], and sealability [4], making it the material of choice in a wide range of endodontic situations.

However, MTA has several limitations, including handling difficulties due to its granular consistency [5], slow setting time, which contributes to leakage, surface disintegration, loss of marginal adaptation, and continuity of the material [6], and a tendency to wash out in the presence of excess moisture [7]. Additionally, discoloration can hinder its full potential. The most significant challenge with MTA is its susceptibility to washout in areas where it comes into contact with blood and tissue fluids.

These problems are further exacerbated in cases of perforation/furcation repair or as a material for root-end filling, and to a lesser extent, in direct pulp capping (DPC) scenarios, where blood, tissue fluids, and serum obstruct the hydration of the material and reduce MTA's microhardness [8,9].

Bone cement has been investigated as a repair material in dentistry, although it has been used in oral and orthopedic surgery for the past 40 years. Bone cement possesses numerous characteristics that make it suitable for a variety of endodontic treatments, including good strength and load-bearing capacity [10,11], favorable handling and working properties, faster setting times of around 15 minutes, tolerance to a moist environment [12], good marginal adaptation as a root-end filling material, and low cytotoxicity [13-15].

However, bone cement does not inherently show bioactivity, which is a crucial property for repair materials. Research has demonstrated that the bioactivity of bone cement can be enhanced by incorporating bioactive materials such as amorphous calcium phosphate, hydroxyapatite (HA), tetracalcium phosphate, and bioactive glass [10,11].

In a previous study by the current author, bioactive bone cement (BBC) was developed by modifying the polymer/powder component of bone cement with MTA. This modification aimed to retain all the favorable properties of bone cement while overcoming the potential drawbacks associated with MTA [15]. This novel BBC is proposed to provide a seal comparable to that of MTA in clinical scenarios such as furcation repair while offering better handling and faster setting times [15]. Studies testing this modified cement for marginal adaptation and sealing ability in the presence of blood and artificial saliva, with results indicating that BBC performed better than biodentine and was comparable to Bio C Repair [16,17]. Additionally, another study comparing push-out bond strength in retrograde cavity preparation showed that BBC had a bond strength comparable to MTA Angelus [18].

However, the bioactivity of this modified cement remains to be tested. This preliminary in vitro study aims to characterize the interactions of BBC with a synthetic tissue fluid composed of a neutral phosphate-buffered saline (PBS) solution using inductively coupled plasma-atomic emission spectroscopy.

## Materials and methods

Study design and setting

This study was an in vitro investigation carried out at the Department of Conservative Dentistry and Endodontics, Sree Balaji Dental College and Hospitals, Chennai, India. It aimed to evaluate the bioactivity of modified bone cement. The research was approved by the Institutional Ethics Committee of Sree Balaji Dental College and Hospital (Ref No. SBDCH - IEC/23-08/44).

Preparation of BBC

Modification of polymethyl methacrylate (PMMA) bone cement (Simplex P, Stryker Corporation, Mahwah, NJ) with alkoxysilanes and calcium salts can impart apatite-forming ability to the cement [19]. The BBC was formulated using a methodology similar to that employed in a previous study [15]. The BBC powder was prepared by combining pre-weighed 0.4 g of MTA with 0.6 g of bone cement (60:40) until all the MTA particles were homogeneously incorporated into the polymer powder. The liquid component was altered by adding one drop of silane coupling agent (Monobond-S) to 1 mL of monomer liquid and mixing them together.

Preparation of samples

Twenty cylindrical plastic molds of 2 mm diameter and 3 mm height were prepared. They were divided into two groups: group 1 (n=10), filled with MTA (ProRoot Mineral Trioxide Aggregate, DENTSPLY, Tulsa Dental Specialties, Tulsa, Oklahoma), and group 2 (n=10), filled with BBC. In group 1, slurry samples of MTA were prepared using 0.25 g of MTA and 1 mL of distilled water and were placed into the cylindrical molds. Each sample was then individually suspended in 2 mL of stimulated tissue fluid (STF) in a transparent glass vial with a rubber stopper. For group 2 samples, the powder and liquid of the modified bone cement were mixed in a 2:1 ratio under ambient conditions at room temperature to obtain a dough-like consistency, which was then packed into the cylindrical molds. These samples were also suspended individually in 2 mL of STF in a transparent glass vial with a rubber stopper. All samples were stored at 37°C. The STF was a phosphate-buffered saline solution (pH=7.2) with the following The data collected were organized and analyzed using Statistical Product and Service Solutions (SPSS, version 24; IBM Corp, Armonk, NY). Initially, a one-way ANOVA was conducted to assess the release of cations across the two groups. Subsequently, an independent sample t-test was performed to compare the cation release between the groups. Statistical significance was determined using a threshold p-value of less than 0.05 composition: 1.7 g of KH2PO4, 11.8 g of Na2HPO4, 80.0 g of NaCl, and 2.0 g of KCl in 10 L of H2O.

Within one to two hours of storage, white precipitates began to form on the surface of both groups of samples, as well as in the surrounding solutions. After seven days of soaking in STF, the set samples were removed from their respective vials to examine the amount of calcium and phosphate ions released from the MTA and the BBC using inductively coupled plasma-optical emission spectroscopy (Optima 2100 DV, PerkinElmer). The amount of calcium and phosphate ions released was measured in parts per million (ppm).

Statistical analysis

The data collected were organized and analyzed using SPSS. One-way ANOVA (analysis of variance was used to assess the release of cations across the two groups. Subsequently, an independent sample t-test was performed for pairwise comparison between the experimental groups. Statistical significance was determined using a p-value threshold of <0.05.

## Results

The study used 20 cylindrical plastic molds, which were divided into two groups based on the type of cement evaluated for bioactivity: MTA and BBC. Table 1 presents the mean concentrations (± SD, ppm) of cations leached from group 1 (MTA) and group 2 (BBC) into simulated tissue fluid after seven days of storage, as measured by atomic spectroscopy. The concentration of calcium ions released was significantly higher compared to phosphate ions in both group 1 (MTA) and group 2 (BBC).

**Table 1:** Group 1 (MTA) and group 2 (BBC) mean values of phosphate ions released obtained through inductively coupled plasma atomic spectroscopy. Ca - Calcium ions; P - Phosphate ions

Groups	Cations released	Mean (ppm)	Standard deviation	Std. Error Mean
Group 1	Ca	41.411	2.483	0.78534
	P	2.379	1.341	0.42391
Group 2	Ca	23.485	2.098	0.66354
	P	7.825	1.402	0.44323

**Figure 1:**
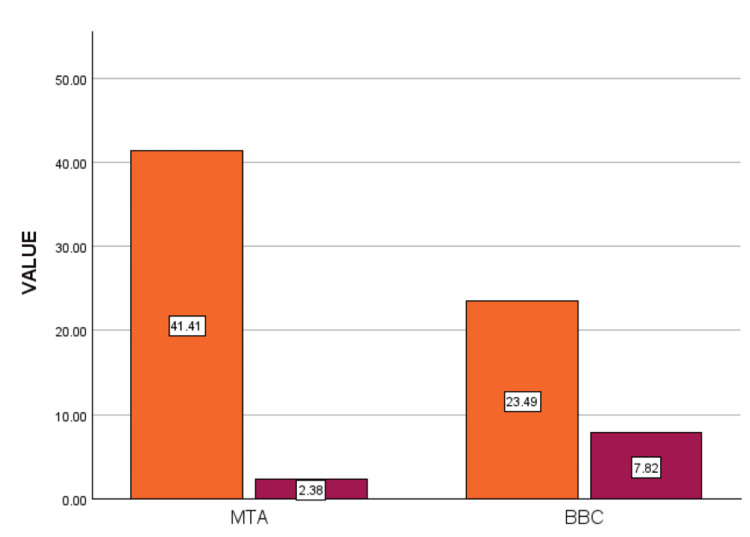
Comparison of calcium and phosphate ions release in group 1 (MTA) and group 2 (BBC). MTA - mineral trioxide aggregate; BBC - bioactive bone cement

As shown in Table 1 and illustrated in Figure 1, both MTA and Bioactive Bone Cement undergo dissolution after one week in simulated tissue fluid, releasing their principal cationic constituents. Figure 1 provides a comparative analysis of calcium and phosphate ion release between the two groups.

Table 2 displays the results of an independent sample t-test comparing calcium ion release between group 1 (MTA) and group 2 (BBC) in simulated tissue fluid, conducted using SPSS. The analysis reveals a statistically significant difference (p < 0.001), indicating that calcium ion release was higher in group 1 (MTA) compared to group 2 (BBC), with a 95% confidence interval.

**Table 2:** Intergroup comparison of calcium release.

Independent Samples Test											
		Levene's Test for Equality of Variances		t-test for Equality of Means							
		F	Sig.	t	df	Significance		Mean Difference	Std. Error Difference	95% Confidence Interval of the Difference	
						One-Sided p	Two-Sided p			Lower	Upper
Value	Equal variances assumed	0.015	0.903	-8.879	18	<0.001>	<0.001>	-5.44560	0.61332	-6.73413	-4.15707
	Equal variances not assumed	NA	NA	-8.879	17.964	<0.001>	<0.001>	-5.44560	0.61332	-6.73431	-4.15689

Table 3 presents the results of an independent sample t-test comparing phosphate ion release between group 1 (MTA) and group 2 (BBC), also conducted using SPSS. This analysis demonstrates a statistically significant difference (p < 0.001), with group 2 (BBC) showing greater phosphate ion release compared to group 1I (MTA), with a 95% confidence interval.

**Table 3:** Intergroup comparison of phosphate release.

Independent Samples Test											
		Levene's Test for Equality of Variances		t-test for Equality of Means							
		F	Sig.	t	df	Significance		Mean Difference	Std. Error Difference	95% Confidence Interval of the Difference	
						One-Sided p	Two-Sided p			Lower	Upper
Value	Equal variances assumed	0.440	0.516	17.436	18	< 0.001>	< 0.001>	17.92600	1.02812	15.76600	20.08600
	Equal variances not assumed	NA	NA	17.436	17.512	< 0.001>	< 0.001>	17.92600	1.02812	15.76167	20.09033

## Discussion

The current study assessed the bioactivity of modified bone cement by evaluating the precipitates formed after placing it in PBS using inductively coupled plasma-atomic spectroscopy.

Within one to two hours of storage, white precipitates appeared on the surface of MTA (group 1) samples and in the surrounding solutions. After 24 hours in PBS, whitish precipitates were formed on the surface of the modified bone cement samples and in the surrounding solutions.

Data obtained through inductively coupled plasma-atomic spectroscopy suggest that both MTA (group 1) and BBC (group 2) dissolve in PBS and release their primary cationic components.

Characterization of these ions revealed the presence of calcium and phosphate in significant quantities in both groups (see Table 1). These results align with the findings of LeGeros [20]. After a seven-day storage period in PBS, both groups showed the release of cations (p > 0.001). The release of calcium ions was higher in MTA (group 1), while the release of phosphate ions was more pronounced in BBC (group 2) (see Tables 2-3).

Among the released ions, calcium stands out as the most predominant due to its limited solubility in biological fluids, resulting in HA precipitation. The specific reaction responsible for this precipitation is as follows:

​10Ca^2+^+6(PO_4_​)^3−^+2(OH)^−^→Ca10​(PO_4_​)^6^​(OH)^2​^

Hydroxyapatite formation can be observed on the surface of bioactive materials in simulated body fluid with ion concentrations nearly equal to human body plasma [21,22]. This biological calcification process is favored at a pH of 7, which aligns with the pH of the simulated body fluid used in this study. This reaction occurs when various calcium-containing materials come into contact with biological environments [23-25]. Fundamental studies concerning the reactions of bioactive glasses and glass ceramics when exposed to simulated body fluid show that apatite formation can be stimulated.

This formation is triggered by the release of calcium ions (Ca²⁺) from the material into the body's fluid environment and is facilitated by the catalytic effect of Si-OH groups that develop on the material's surface. This suggests that the bioactivity of a material depends on the presence of silane groups and calcium salts. Introducing Si-OH groups and calcium ions could render bone cement bioactive. Calcium salts initiate the formation of hydroxyapatite (HaP), while Si-OH groups are responsible for the deposition of this triggered HaP through the process of heterogeneous nucleation of the apatite layer in the body environment [26].

Based on this, we developed bioactive bone cement by modifying the liquid component of bone cement with a silane coupling agent (MPS), which provides Si-OH groups due to hydrolysis of alkoxysilane upon exposure to the body environment. The powder component was modified with MTA to provide calcium salts. MTA was chosen for modifying the powder component due to its established role as a repair material with favorable sealing ability, bioactivity, and biocompatibility arising from its physicochemical interactions with tissues [20].

The role of the silane coupling agent is multifold: it accelerates apatite formation through heterogeneous nucleation, thereby reducing the amount of calcium salts needed to modify the bone cement while maintaining the mechanical properties of the cement. It also increases the compressive strength of the modified material by forming a chemical bond between the filler MTA particles and polymerized bone cement [26].

Bone cement has been utilized for localized drug delivery in orthopedics. It has been used for the targeted delivery of growth factors, along with compounds that have anti-inflammatory, anticancer, analgesic, and antibiotic properties, among other therapeutic additives [27,28]. For example, a study presented gentamicin-loaded bone cement as a root-end and bone defect-filling material in apical surgery cases [29]. BBC could thus act as a targeted delivery system for MTA while facilitating placement in areas with excess blood or tissue fluids.

The utilization of bone cement, even in the substantial quantities required for total hip arthroplasty, appears to be devoid of adverse effects. This assertion is supported by the literature as well as two decades of surgical practice. The volumes needed for orthopedic applications significantly surpass those required in endodontics. Therefore, the transition of bone cement from medical to dental use may be achieved with relative ease. Smaller quantities necessary in dentistry would result in a considerably lower exothermic reaction and a reduced amount of free monomer. Based on the results of the current study, it can be clearly concluded that BBC (group 2) retains its bioactivity when it comes into contact with a stimulated oral environment (STF). This demonstrates that BBC is bioactive in a simulated oral environment.

The limitation of this study is its focus on a single variable, namely, bioactivity. Evaluating additional factors, such as pushout bond strength and resistance to washout, could offer valuable clinical insights, especially since bioactivity has already been demonstrated. Moreover, performing an SEM analysis of the interface between the BBC and dentin could provide further information on the nature of the bond formed between the cement and dental hard tissues.

## Conclusions

Within the limitations of the study, it can be concluded that the BBC exhibited a release of calcium and phosphate comparable to that of MTA when placed in a PBS solution. Although the release of calcium was lower than that of MTA, the modified cement retained the favorable handling properties of bone cement and could be easily manipulated into a dough form. Clinically, in cases of apical surgery, internal resorption, or perforation repair where material placement is challenging, BBC can prove to be beneficial as a localized MTA delivery system.
